# Outcomes of patients with acetaminophen-associated toxic hepatitis at a far east poison center

**DOI:** 10.1186/2193-1801-2-674

**Published:** 2013-12-17

**Authors:** Yi-Chou Hou, Ja-Liang Lin, Wen-Hung Huang, Cheng-Hao Weng, Shen-Yang Lee, Ching-Wei Hsu, I-Kuan Wang, Chih-Chia Liang, Chiz-Tzung Chang, Wey-Ran Lin, Tzung-Hai Yen

**Affiliations:** Department of Nephrology and Division of Clinical Toxicology, Chang Gung Memorial Hospital and Chang Gung University, Taipei, Taiwan; Department of Nephrology, China Medical University Hospital and China Medical University, Taichung, Taiwan; Department of Gastroenterology and Hepatology, Chang Gung Memorial Hospital and Chang Gung University, Taipei, Taiwan; Department of Nephrology, Chang Gung Memorial Hospital, 199 Tung Hwa North Road, Taipei, 105 Taiwan

**Keywords:** Acetaminophen, Poisoning, Suicide, Toxic hepatitis, *N*-acetylcysteine

## Abstract

**Background:**

There is an overall paucity of data regarding the outcomes of patients with acetaminophen-associated toxic hepatitis in Taiwan. Therefore, the purpose of this study was to recruit a larger number of patients and to examine the clinical features, the degrees of toxic hepatitis, the physiological markers, and the clinical outcomes after intentional acetaminophen poisoning, and to determine what association, if any, might exist between these findings.

**Methods:**

We examined the medical records of 187 patients with intentional acetaminophen poisoning who were examined at Chang Gung Memorial Hospital between 2000 and 2011. Patients were categorized into 2 groups according to hepatic complications, i.e. with (n = 15) or without (n = 172) toxic hepatitis. Demographic, clinical, and laboratory data were collected, and the mortality rate was analyzed.

**Results:**

It was found that patients with toxic hepatitis had higher serum acetaminophen level (P = 0.007), but they also arrived to the hospital later (P < 0.001) than patients without toxic hepatitis. Furthermore, patients with toxic hepatitis showed higher incidences of acute respiratory failure (P = 0.012) than those shown by patients who did not have hepatitis. The laboratory examinations also revealed greater degrees of granulocytosis (P < 0.001) and poorer liver function tests (P < 0.001) in patients with hepatitis than in patients without hepatitis. Nevertheless, a univariate logistic regression model failed to identify any significant risk factors for toxic hepatitis complication after ingestion (P > 0.05). At the end of the analysis, 1 patient with toxic hepatitis died of liver failure. Finally, there was no significant difference in mortality between patients with and without hepatitis (P = 0.080).

**Conclusion:**

The analytical data revealed that toxic hepatitis was not uncommon (15/187 or 8.0%) after acetaminophen overdose. Further studies are warranted.

## Introduction

Acetaminophen is one of the best-selling analgesics in Taiwan and one of the most popular drugs in the world. Although it is safe at therapeutic doses, overdoses of acetaminophen can cause severe liver injury. Although the first case of acetaminophen-related hepatic necrosis due to overdose was documented in 1966 (Davidson and Eastham, 1966), it is still the leading agent of liver failure due to poisoning (Lee, 2004).

Acetaminophen is ingested orally. The daily maximum dose is 4 g for an adult and 80 mg/kg for a child. After ingestion, the concentration peaks around 2 h. Dosages that induce toxic hepatitis are greater than 12 g per day in adults and 250 mg/kg over 24 h in children (Makin et al., 1995). After ingestion, around 90% of the acetaminophen is metabolized to sulfate and glucuronide, which is conjugated in the liver and then excreted into the urine. Eight percent of the remaining acetaminophen is metabolized by cytochrome p450 into the more toxic form of *N*-acetyl-*p*-benzoquinoneimine (NAPQI) when the glutathione (GSH) is depleted (Bessems and Vermeulen, 2001). NAPQI then binds to the cysteine group in the hepatocyte, and oxidative injury and hepatocellular centrilobular necrosis is then induced (Jollow et al., 1973). However, *N*-acetylcysteine limits the toxicity of acetaminophen by increasing the GSH stores, binding with NAPQI as a substitute for GSH, and enhancing sulfate conjugation (Lee, 2004).

There is an overall paucity of data regarding the outcomes of patients with acetaminophen poisoning in Taiwan. In the only original research study, Tsai et al. (Tsai et al., 2004) have reported that age and the time to presentation were the independent risk factors for hepatotoxicity (P = 0.033 and P = 0.002, respectively) after acetaminophen intoxication. However, due to their low sample size, the certainty of the conclusions may be limited.

Therefore, the purpose of this study was to recruit a larger number of patients and to examine the clinical features, the degrees of toxic hepatitis, the physiological markers, and the clinical outcomes after intentional acetaminophen poisoning, and to determine what association, if any, might exist between these findings.

## Materials and methods

### Ethics statement

This retrospective observational study complied with the guidelines of the Declaration of Helsinki and was approved by the Medical Ethics Committee of Chang Gung Memorial Hospital, which is a tertiary referral center located in the northern part of Taiwan. Because this study involved a retrospective review of existing data, the Institutional Review Board approval was obtained, but specific informed consents were not obtained from the patients. However, informed consents of the risks of acute acetaminophen poisoning and all treatment modalities were obtained from all patients upon their initial admission. In addition, all individual information was securely protected by delinking identifying information from the main data set and available to investigators only. Furthermore, all of the data were analyzed anonymously. The Institutional Review Board of Chang Gung Memorial Hospital specifically waived the need for consent. Finally, all primary data were collected according to the Strengthening the Reporting of Observational Studies in Epidemiology guideline. The policy was based on previous publication (Liu et al., 2012; Chen et al., 2013, Yu et al., 2013).

### Patients

We examined the records of 187 patients with intentional acetaminophen poisoning who were examined at Chang Gung Memorial Hospital between 2000 and 2011. Demographic, clinical, and laboratory data were collected, and the mortality rate was analyzed. The diagnoses of acetaminophen intoxication were based on clinical, physical and laboratory examinations, and confirmed by plasma acetaminophen levels (fluorescence polarization immunoassay).

### Inclusion and exclusion criteria

All patients who were older than 18 years of age and who were diagnosed with acetaminophen poisoning at Chang Gung Memorial Hospital between 2000 and 2011 were eligible for inclusion in this study. Patients were excluded if they were younger than 18 years or did not have detectable acetaminophen levels in the blood despite a positive history of ingestion or if they had major systemic comorbidities, such as cancer or heart, lung, renal, or liver diseases.

### Detoxification protocol

The protocols that were used to treat patients included gastric lavage with large amounts of normal saline, which was followed by the infusion of 1 g/kg activated charcoal and 250 mL magnesium citrate through a nasogastric tube. Magnesium citrate was used to prevent constipation after the charcoal administration. The use of the *N*-acetylcysteine antidote was determined with toxicity estimations from the Rumack-Matthew nomogram for single acute acetaminophen overdose/ingestion (Wolf et al., 2007).

### Definitions of clinical events

Acetaminophen related toxic hepatitis was diagnosed if the alanine aminotransferase (ALT) increase was more than 1000 U/L (McClain et al., 1999; Vale and Proudfoot, 1995; Ayonrinde et al., 2005). Hepatitis B virus carrier was defined as positive hepatitis B surface antigen in the blood of more than 6 months. Acute renal failure was defined as serum creatinine levels over 1.3 mg/dL (Weng et al., 2012). Acute respiratory failure has been defined as a condition of respiratory insufficiency requiring intubation and mechanical ventilation for more than 24 h, regardless of the fraction of inspired oxygen (Luhr et al., 1999).

### Statistical analysis

The continuous variables are expressed as means and standard deviations, and categorical variables are expressed as numbers with percentages in brackets. All data were tested for the normality of distribution and the equality of standard deviations before the analysis. For comparisons between patient groups, we used Student’s *t*-tests for the quantitative variables and Chi-square or Fisher’s exact tests for the categorical variables. An initial univariate Cox regression analysis was performed in order to compare the frequencies of the possible risk factors that were associated with toxic hepatitis complication. In order to control for possible confounding factors, a multivariate Cox regression analysis (stepwise backward approach) was performed in order to analyze those factors that were significant in the univariate models (P < 0.05) and that met the assumptions of a proportional hazard model. We considered the results that rejected the null hypothesis with 95% confidence to be significant. All analyses were performed with the IBM SPSS Statistics Version 20 software program (IBM Corporation, Armonk, NY, USA).

## Results

Table 1 outlines the baseline characteristics of the 187 patients with acetaminophen poisoning who were grouped according to hepatitis complications. It was found that patients with toxic hepatitis had higher serum acetaminophen level (P = 0.007) and they also arrived to the hospital later (P < 0.001) than patients without toxic hepatitis. Notably, the prevalence of hepatitis B virus carrier was also higher in patients with than patients without toxic hepatitis (P < 0.001).

**Table 1:** **Baseline characteristics of patients with acetaminophen poisoning (N = 187)**

Variable	Total (N = 187)	No toxic hepatitis (N = 172)	Toxic hepatitis (N = 15)	P
Serum acetaminophen (μg/mL)	57.6 ± 60.3	114.4 ± 98.6	238.1 ± 300.0	0.007**
Acetaminophen amount (tablet, 500 mg per tablet)	37.1 ± 25.2	36.5 ± 25.1	44.2 ± 27.0	0.973
Age (years)	28.9 ± 12.1	29.0 ± 12.4	28.4 ± 8.3	0.550
Male, n (%)	35 (18.7)	31 (18.0)	4 (26.6)	0.300
Time elapsed between acetaminophen ingestion and hospital arrival (hour)	6.2 ± 6.0	5.4 ± 4.1	16.1 ± 13.5	<0.001***
Hepatitis B virus carrier, n (%)	10 (5.3)	6 (3.5)	4 (26.6)	<0.001***
Alcohol consumption, n (%)	35 (18.7)	32 (18.6)	3 (20.0)	0.599
Smoking habit, n (%)	29 (15.5)	26 (15.1)	3 (20.0)	0.420
Hypertension, n (%)	6 (3.3)	5 (2.9)	1 (6.7)	0.399
Diabetes mellitus, n (%)	3 (1.6)	3 (1.7)	0 (0)	0.777
Malignancy, n (%)	3 (1.6)	3 (1.7)	0 (0)	0.777
Depressive disorder, n (%)	125 (66.8)	114 (66.2)	11 (73.3)	0.404
Amphetamine abuse, n (%)	1 (0.5)	1 (0.7)	0 (0)	0.607
Ketamine abuse, n (%)	2 (1.1)	1 (0.7)	0 (0)	0.308
Heroin abuse, n (%)	0 (0)	0 (0)	0 (0)	1.000
Use of benzodiazepine hypnotics, n (%)	40 (21.4)	38 (22.1)	2 (13.3)	0.338

Note: **P < 0.01, ***P < 0.001.

As shown in Table 2, patients with toxic hepatitis showed higher incidences of acute respiratory failure (P = 0.012) than those shown by the patients who did not have hepatitis. The laboratory examinations revealed greater degrees of granulocytosis (P < 0.001) and poorer liver function tests (P < 0.001) in patients with hepatitis than in patients without hepatitis (Table 3).

**Table 2:** **Clinical manifestations of patients with acetaminophen poisoning (N = 187)**

Variable	Total (N = 187)	No toxic hepatitis (N = 172)	Toxic hepatitis (N = 15)	P value
Vital signs				
Systolic blood pressure (mmHg)	120.8 ± 18.8	120.1 ± 17.8	127.9 ± 26.1	0.008**
Diastolic blood pressure (mmHg)	72.6 ± 13.3	72.3 ± 13.0	75.9 ± 16.0	0.301
Heart rate (beat per minute)	82.1 ± 16.4	81.9 ± 16.1	83.4 ± 20.0	0.494
Body temperature (°C)	36.8 ± 4.4	36.8 ± 4.6	36.9 ± 0.84	0.718
Urinary system				
Acute renal failure, n (%)	0 (0)	0 (0)	0 (0)	1.000
Gastrointestinal system				
Nausea and vomiting, n (%)	81 (43.3)	71 (41.2)	10 (66.6)	0.052
Neurologic system				
Conscious disturbance, n (%)	41 (21.9)	38 (22.0)	3 (20.0)	0.575
Headache, n (%)	8 (4.3)	7 (4.1)	1 (5.1)	0.768
Seizure, n (%)	2 (1.1)	1 (0.01)	1 (5.1)	0.154
Respiratory system				
Acute respiratory failure, n (%)	7 (3.7)	4 (2.3)	3 (20.0)	0.012*

Note: *P < 0.05, **P < 0.01.

**Table 3:** **Laboratory data of patients with acetaminophen poisoning (N = 187)**

Variable	Total (N = 187)	No toxic hepatitis (N = 172)	Toxic hepatitis (N = 15)	P value
First day (arrival)				
Hemoglobin (g/dL)	13.4 ± 1.5	13.4 ± 1.5	13.6 ± 1.3	0.589
Neutrophils (/μL)	9078.7 ± 4077.6	8747.5 ± 3271.6	10305.4 ± 6123.8	<0.001***
Platelet (10^3^/μL)	246.3 ± 68.9	250.9 ± 61.9	196.5 ± 111.7	0.006
Urea nitrogen (mg/dL)	8.8 ± 4.5	8.9 ± 4.6	7.6 ± 3.4	0.542
Creatinine (mg/dL)	0.8 ± 0.2	0.7 ± 0.2	0.8 ± 0.3	0.060
Sodium	140.1 ± 3.2	140.2 ± 3.2	139.1 ± 2.5	0.180
Potassium	3.6 ± 1.6	3.6 ± 1.7	3.3 ± 0.4	0.584
AST (U/L)	146.1 ± 1125.7	31.1 ± 34.4	1497.6 ± 3915.7	<0.001***
ALT (U/L)	75.4 ± 380.6	23.6 ± 31.9	720.7 ± 1264.8	<0.001***
Total bilirubin (mg/dL)	1.0 ± 0.5	0.9 ± 0.4	1.7 ± 1.0	<0.001***
Albumin (g/dL)	4.0 ± 0.7	4.1 ± 0.6	3.6 ± 0.7	0.588
Prothrombin time (INR, sec)	1.2 ± 0.4	1.1 ± 0.1	2.0 ± 0.9	0.000***
γ-glutamyl transferase (U/L)	75.6 ± 107.3	39.6 ± 61.5	290.1 ± 16.3	0.327
Alkaline phosphatase (U/L)	51.6 ± 19.2	47.7 ± 16.4	70.1 ± 21.9	0.433
Ammonia	81.0 ± 67.6	54.5 ± 29.6	137.6 ± 92.0	0.010
Second day				
AST (U/L)	503.1 ± 2005.0	37.4 ± 75.9	3962.7 ± 4637.8	<0.001***
ALT (U/L)	377.0 ± 1254.8	47.1 ± 117.2	3148.5 ± 2532.4	<0.001***
Total bilirubin (mg/dL)	1.2 ± 1.3	0.9 ± 0.6	2.9 ± 2.4	<0.001***
Prothrombin time (INR, sec)		1.1 ± 0.1	2.4 ± 0.8	<0.001***
Fourth day				
AST (U/L)	302.4 ± 931.7	35.5 ± 63.2	1575.4 ± 1791.3	<0.001***
ALT (U/L)	343.0 ± 1035.4	56.4 ± 126.4	2745.4 ± 1915.3	<0.001***
Total bilirubin (mg/dL)	0.9 ± 0.8	0.7 ± 0.5	1.8 ± 1.3	<0.001***
Prothrombin time (INR, sec)		1.1 ± 0.1	1.9 ± 0.8	<0.001***

Note: ***P < 0.001.

Table 4 shows that most patients were treated aggressively with gastric lavage, which was followed by an infusion of activated charcoal and magnesium citrate. In addition, most patients were treated with the *N*-acetylcysteine antidote. Nevertheless, patients with toxic hepatitis received lesser proportion of gastric lavage and active charcoal than patients without toxic hepatitis (P = 0.002).

**Table 4:** **Treatment and outcome of patients with acetaminophen poisoning (N = 187)**

Variable	Total (N = 187)	No toxic hepatitis (N = 172)	Toxic hepatitis (N = 15)	P value
Gastric lavage, n (%)	122 (65.2)	118 (68.6)	4 (26.7)	0.002**
Active charcoal and magnesium citrate, n (%)	122 (65.2)	118 (68.6)	4 (26.7)	0.002**
N-acetylcysteine, n (%)	147 (78.6)	133 (77.3)	14 (93.3)	0.212
Mortality, n (%)	1 (0.5)	0 (0)	1 (6.7)	0.080

Note: **P < 0.01.

At the end of the analysis, 1 patient with toxic hepatitis died of liver failure (Table 4). Nevertheless, there was no significant difference in mortality between patients with and without hepatitis (P = 0.051).

Finally, a univariate logistic regression analysis failed to identify any significant risk factors for toxic hepatitis complication after ingestion (Table 5).

**Table 5:** **Clinical predictors of toxic hepatitis after acetaminophen poisoning (N = 187)**

Variable	Univariate logistic analysis		
	Odds ratio	95% confidence interval	P value
Serum acetaminophen level (each increase of 1 μg/mL)	1.006	0.997-1.016	0.210
Acetaminophen amount (each increase of 1 tablet)	1.039	0.984-1.096	0.169
Hepatitis B virus carrier (yes)	0.000	0.000-0.000	0.999
Time elapsed between acetaminophen ingestion and hospital arrival (each increase of 1 hour)	1.260	0.241-9.438	0.660

## Discussion

Acetaminophen is a well-known agent that causes dose-dependent toxic hepatitis. It was revealed in this study that although patients with toxic hepatitis suffered higher serum acetaminophen levels than patients without toxic hepatitis, a univariate logistic regression analysis failed to identify serum acetaminophen level as a significant risk factor for toxic hepatitis complication after ingestion, possibly due to small sample size and short follow up duration (Table 5). Theoretically, the toxic metabolite NAPQI is formed by cytochrome p450 and then detoxified by conjugating with GSH (Nelson, 1990), and oversaturated NAPQI makes the hepatocytes more vulnerable to injury (Potter et al., 1973). Nevertheless, the amount of acetaminophen in patients without toxic hepatitis in was 36.5 ± 25.1 tablets (or 17.8 ± 12.6 g), which was much higher than 4 g, the maximum recommended dose for adult (Table 1). Mitochondria injury may be the major determinant for this because previous animal studies have demonstrated that NADQI binds to mitochondria and leads to oxidative stress (Jaeschke, 1990). NADQI induced mitochondria permeability transition pore opening and mitochondrial permeability changes (Bajt et al., 2006). Endonuclease G and the apoptosis-inducing factor are released by mitochondria, and DNA fragmentation occurs with the release of these enzymes (Kon et al., 2004). Although oversaturation is the major mechanism in acetaminophen-related toxic hepatitis, mitochondria-related cytotoxic damage may be the reason for the heterogeneity of the phenotypes after the ingestion of large amounts of acetaminophen.

In this study, 1 patient with toxic hepatitis died of liver failure, and the overall mortality rate was 0.5% (Table 4). Evidently, the favorable outcomes depended on a prompt diagnosis of acetaminophen poisoning and the immediate institution of detoxification protocols. In US reports on acetaminophen-related acute hepatitis and liver failure, the mortality rate has been reported to be around 19–30% (Lee, 2008; Schiodt et al., 1997). Larson et al. (Larson et al., 2005) have reported that 27% of US patients with acetaminophen-induced acute liver failure died in 3 weeks. Therefore, our mortality rate was much lower than the data from the US Poison Centers. In terms of metabolism (Patel et al., 1992), acetaminophen is extensively conjugated with glucuronic acid and sulfate prior to renal excretion. A minor metabolic route involves the microsomal oxidation of acetaminophen to a hepatotoxic reactive intermediate, which subsequently undergoes GSH conjugation, yielding cysteine and mercapturate conjugates, both of which are excreted in the urine. In a previous pharmacogenetic study (Patel et al., 1992), it has been reported that the mean fractional excretion of cysteine metabolites differed between Caucasians and Orientals (P < 0.005). Therefore, it is unclear whether the low mortality rate in our study can be explained by ethnic differences in enzymatic metabolism between Orientals and Caucasian.

The prevalence of hepatitis B virus carrier in Taiwan is around 15–20%, which is higher than in the Europe or US (Ott et al., 2012; Lee et al., 2013). The percentage of the hepatitis B virus carrier in our study was 5.3% (Table 1), which was much lower than national Taiwanese registry (Lee et al., 2013). Nevertheless, the prevalence of hepatitis B virus carrier was higher in patients with than patients without toxic hepatitis (Table 1). Although the presence of hepatitis B carrier was not longer a significant predictor for toxic hepatitis after logistic regression analysis (Table 5), it was the authors’ thought that the viral carrier status might make hepatocytes more vulnerable to toxic injury. Literature data on the influence of acetaminophen intake on acute viral hepatitis is scarce. It was disclosed in a small clinical study (Yaghi et al., 2006) that the use of acetaminophen at therapeutic doses was associated with greater alterations of surrogate markers of the severity of acute viral hepatitis. On the other hand, susceptibility to acetaminophen toxicity was unexpectedly decreased during acute viral hepatitis in mice (Getachew et al., 2010). Therefore, further studies should be considered to confirm if infected hepatocytes are more vulnerable to acetaminophen toxicity.

It was revealed in this study that the onset of toxic hepatitis was within hours after acetaminophen exposure (Table 3), which was in sharp contrast to our previous experience with paraquat-induced toxic hepatitis (Yang et al., 2012). In paraquat patients, the symptoms of toxic hepatitis usually developed within 6.7 ± 6.3 days of exposure, peaked at 9.5 ± 8.8 days, and were resolved in 17.3 ± 9.8 days. It was hypothesized by Mullick et al. (Mullick et al., 1981) that paraquat injury to the liver is biphasic; it is initially hepatocellular but becomes cholangiocellular after the first 2 days. However, only hepatocellular damage mechanisms have been implicated following acetaminophen intoxication (Larson, 2007). Therefore, it is unclear whether the faster onset of toxic hepatitis in cases of acetaminophen poisoning can be explained by different liver injury mechanisms.

In this study, patients with toxic hepatitis received lesser proportion of gastric lavage and active charcoal than patients without toxic hepatitis (Table 4). This could be due to longer time elapse between acetaminophen ingestion and hospital arrival in patients with than without toxic hepatitis (Table 1). Similarly, the time elapse was not a significant risk factor for mortality after logistic regression analysis (Table 5). In a study that was conducted in Hong Kong (Chan et al., 1996), it was found that the time that elapsed between acetaminophen ingestion and treatment was the most important prognostic factor for liver toxicity. However, the *N*-acetylcysteine antidote was only used in 50% of the patients (Chan et al., 1996), but the proportion of patients that received *N*-acetylcysteine treatment was as high as 78.6% in our study (Table 4). Therefore, it is unclear whether the difference in the use of the *N*-acetylcysteine antidote could influence predictions of time elapses for toxic hepatitis.

The analytical results showed that patients with toxic hepatitis showed higher incidences of acute respiratory failure than those shown by the patients who did not have hepatitis (Table 2). The results of a laboratory study (Ilic et al., 2010) have also indicated that a high hepatotoxic dose of paracetamol (5 g per kg intraperitoneally) produced hepatic encephalopathy with generalized convulsions in rats. Hepatic coma by liver failure may be possible cause inducing respiratory failure or high anion gap metabolic acidosis (Mendoza et al., 2006).

In summary, the analytical data revealed that toxic hepatitis was not uncommon (15/187 or 8.0%) after acetaminophen overdose. Nevertheless, this study failed to identify any significant risk factors for mortality, possibly due to low sample size and short follow up duration. Further studies are warranted.
